# THE CAMARADERIE OF MIDDLE-AGED AND OLDER WOMEN: HOW DOES FLASH MOB BUILD SOCIAL CAPITAL?

**DOI:** 10.1093/geroni/igae098.2433

**Published:** 2024-12-31

**Authors:** Hae Youn Min, Kyung Eun Lee, Jinmoo Heo

**Affiliations:** Yonsei University, Seoul, Republic of Korea; Yonsei University, Seoul, Republic of Korea; Yonsei University, Seoul, Republic of Korea

## Abstract

The “flash mob”, a new indication of 21st-century digital culture, involves groups of unfamiliar individuals gathering suddenly in public spaces to perform distinctive activities based on pre-arranged instructions and dispersing quickly. Engaging in group events like flash mobs enhances a more profound sense of community, belonging, and friendship. This study explores the potential of flash mobs to encourage social capital, focusing on a specific group of middle-aged and older women who engage in flash mobs as a means of celebrating the aging process. This group of women gathered to welcome the journey of aging, using flash mobs to show the way to enjoy the moment. The study used a qualitative research method, including in-depth interviews with 16 participants. The findings of this study revealed that participants formed a vivid network, and the outcomes of the network are as follows: (a) strong trust, (b) reciprocity, (c) camaraderie, and (d) contribution to the community. These elements are related closely to Putnam’s (1995) conceptualization of social capital, demonstrating the unique role of flash mobs in impacting social networks and community bonds among participants. Primarily middle-aged and older women, during significant life transitions like empty nest syndrome, retirement, and menopause, they often face loneliness, emphasizing the role of social activity. This study provides insights into how the social capital of middle-aged and older women enhances the anticipation of later life.

